# Diagnosis of Lung Diseases in the Artificial Intelligence Era

**DOI:** 10.3390/ijms27041796

**Published:** 2026-02-13

**Authors:** Kostas A. Papavassiliou, Amalia A. Sofianidi, Athanasios G. Papavassiliou

**Affiliations:** 1 First University Department of Respiratory Medicine, ‘Sotiria’ Chest Hospital, Medical School, National and Kapodistrian University of Athens, 11527 Athens, Greece; 2 Department of Biological Chemistry, Medical School, National and Kapodistrian University of Athens, 11527 Athens, Greece; amaliasof@med.uoa.gr

Artificial intelligence (AI) has revolutionized every field of medicine. By enhancing diagnostic accuracy, accelerating drug discovery, and enabling personalized treatment approaches, AI will soon possess a predominant role as a clinical decision support system. Pulmonary care is one of its most outstanding applications. From interpreting simple X-rays [1] to analyzing multimodal computed tomography (CT) scans [2] for early disease recognition, AI has transformed into a valuable tool for pulmonary clinicians. Machine learning and deep learning demonstrate high sensitivity and accuracy for thoracic imaging [3]. However, the applications of AI in the diagnosis of lung diseases are not restricted to imaging. Rather, they take the initiative to a higher degree; digital pathology [4], pulmonary function test analysis [5], and even lung auscultation [6] are enabled with the use of AI. It is beyond doubt that AI tools are ameliorating how clinicians diagnose and treat lung maladies with exceptional accuracy.

Lung cancer patients experience delays in diagnosis, impacting patient mortality in a disease that already represents the leading cause of cancer death globally [7]. AI could improve lung cancer diagnosis in the three following fields: radiomics (extraction of a large number of quantitative features (like texture, shape, intensity) from standard medical images), digital pathology, and genomic sequencing. Radiomics can offer insightful pathological data from routine radiology scans (CT, magnetic resonance imaging (MRI)) prior to final pathological confirmation [8]. Deep learning models have been invented to differentiate benign from malignant pleural effusions based solely on CT scans [9]. AI algorithms can also identify histological subtypes of non-small cell lung cancer (NSCLC) using positron emission tomography (PET)/CT images with high accuracy [10]. Other models aim at identifying patients with lung adenocarcinoma (the most common form of NSCLC) who have a higher risk of recurrence or metastasis. By combining radiomic, clinical, pathologic, and genomic features, oncologic outcomes for stage I NSCLC could be easily predicted, ultimately characterizing lung adenocarcinoma subtypes that are more aggressive in nature [11]. AI tools can also identify driver mutations; a machine learning model was able to identify *epidermal growth factor receptor* (*EGFR*)-mutant patients through radiomics analysis [12]. *EGFR* exon 19 deletion (EGFR 19Del) and *EGFR* exon 21 L858R mutation (EGFR L858R) have been detected with the use of this AI model [12]. *Kirsten rat sarcoma virus oncogene homolog* (*KRAS*) mutations in NSCLC can also be predicted with the use of a radiogenomics-machine learning predictive model [13]. Additionally, complex interactions between transcription factors/cofactors and their target genes as well as transcription factor upstream signaling networks could be unraveled with the use of AI tools. Accessing huge databases and offering rapid data processing, AI could delineate signal transduction cascades, reveal proteomic and metabolomic signatures, and perform host microbiome analysis; useful tools in personalized medicine [14]. In terms of immunotherapy, a lung cancer immunotherapy-radiomics prediction vector has been established, successfully predicting programmed death-ligand 1 (PD-L1) positivity in NSCLC patients and thus, assessing patients’ appropriateness for immune checkpoint inhibition therapy [15]. Digital pathology is another field aiding early lung cancer diagnosis.

Transforming cryosectioned to formalin-fixed and paraffin-embedded images [16], editing cytological images from patient samples [17], and using universal systems to assess PD-L1 expression [18] with the use of AI algorithms are strategies that could ease the work of pathologists and minimize the subjectivity of their work. Besides PD-L1 expression levels, biomarkers based on the role of tumor-infiltrating lymphocytes (TILs) in the tumor microenvironment (TME) could prove valuable in predicting the efficacy of immune checkpoint inhibitors (ICIs). AI tools have been developed for analyzing spatial TILs expression and prognosticating tumor response [19]. However, the most exciting application of AI in lung cancer diagnosis comes from genomic sequencing identification. Using AI-assisted technologies could identify somatic mutation signatures with next-generation sequencing (NGS) in lung cancer patients [20]. Additionally, a recently developed machine learning tool helped identify and classify the origin of cancer of unknown primary, using NGS data [21]. In the era of precision oncology, AI is able to determine neoantigens [22] and T-cell receptor-antigen binding specificity [23], guiding immunotherapy responses. Additionally, when it comes to screening high-risk populations for lung cancer, AI’s ability to analyze circulating tumor DNA (ctDNA) from liquid biopsies could uncover genetic mutations associated with lung cancer. The identification of lung cancer-associated signatures could aid in detecting lung cancer at such an early stage, at which not even traditional diagnostic methods can detect the disease [24].

The diagnosis of lung diseases that require invasive procedures could also be eased with AI tools. For example, the gold standard for the diagnosis of pulmonary arterial hypertension (PAH) is right heart catheterization, an invasive procedure frequently associated with diagnostic delays [25]. However, non-invasive AI-assisted screening options have been recently described in the literature. Phonocardiogram analysis revealing auscultation signatures indicative of PAH [26], electrocardiogram (ECG)-based algorithms that detect specific abnormalities observed in PAH [27], and imaging data analysis [28] are potential applications of AI in the diagnosis of this complex disease. Similarly, interstitial lung disease (ILD) is a multifaceted disease that either requires invasive methods for diagnosis or high-level imaging interpretation [29]. Lung auscultation could give early signs of the disease; nevertheless, lung crackles are frequently non-specific and cannot be attributed solely to ILD by physician scientists. AI-based lung auscultation could significantly aid physicians who suspect ILD with non-invasive methods [6]. Indeed, digital analysis of pathological lung sounds has been described with deep learning models, ultimately identifying early fibrotic changes with high accuracy [6]. Another AI algorithm used clinical history data from a national US database to identify clinical features predictive of ILD. Surprisingly, results were also extremely accurate, suggesting that there is room for AI to predict ILD diagnosis [30]. Most recently, a multimodal approach was explored, combining CT and histopathological images to enhance the diagnostic accuracy of pathologists and influence their final report [31].

Obstructive lung diseases have not been excluded from the applications of AI in their diagnosis. In this setting, AI has gone one step further; AI-driven smartphone screening for chronic obstructive pulmonary disease (COPD) or asthma exacerbations. Acute exacerbations of COPD and asthma require urgent intervention. An AI tool has been developed, exploiting smartphone microphones for lung sounds auscultations. High specificity and sensitivity rates were reported, while it was noted that enormous amounts of resources could be saved from unnecessary visits to the emergency departments for non-specific subjective patient-reported symptoms [32]. Similarly, asthma exacerbation symptoms overlap with common cold symptoms and as a result, unawareness is common. Prompt detection could be facilitated with the use of smartphones, wheeze detectors, AI cough monitors and smartwatches [33,34]. Moving to a more molecular and personalized approach, the COPDGene study collected CT images, blood transcriptomics, and detailed phenotype information from patients with COPD to better understand the molecular mechanisms underpinning systemic inflammation [35]. This powerful tool could transform precision medicine, finding potential targets for drug development in the treatment of obstructive lung diseases.

In conclusion, AI-powered tools hold the potential to enhance lung disease diagnostics, specifically when applied at a multimodal level, combining imaging, pathology reports, and clinical examination. In the era of individualized and molecular medicine, their most prosperous application could derive from the identification of gene panels, signaling pathways, and targets for selective and patient-tailored treatment approaches. Even in resource-limited domains, where access to physicians and medical facilities is scarce, AI could improve admittance to healthcare. Indeed, with the use of AI, awareness could be raised for early recognition of severe lung diseases, such as ILD, a lung malady with comparable morbidity rates to cancer. However, it should be highlighted that AI cannot and should not replace skilled physicians. Empathy, physical embodiment, and clinical judgment cannot be replaced by robots. The future lies in a partnership amongst physicians and AI tools, which will help them make medical decisions faster, with greater accuracy, while simultaneously minimizing medical errors.
